# Isolation of Industrial Important Bioactive Compounds from Microalgae

**DOI:** 10.3390/molecules26040943

**Published:** 2021-02-10

**Authors:** Vimala Balasubramaniam, Rathi Devi-Nair Gunasegavan, Suraiami Mustar, June Chelyn Lee, Mohd Fairulnizal Mohd Noh

**Affiliations:** 1 Nutrition, Metabolism & Cardiovascular Research Centre, Institute for Medical Research, NIH, Level 3, Block C7, No. 1, Jalan Setia Murni U13, Setia Alam, Shah Alam 40170, Malaysia; rathidevinair@moh.gov.my (R.D.-N.G.); suraiami@moh.gov.my (S.M.); fairulnizal@moh.gov.my (M.F.M.N.); 2 Herbal Medicine Research Centre, Institute for Medical Research, NIH, Level 3, Block C7, No. 1, Jalan Setia Murni U13, Setia Alam, Shah Alam 40170, Malaysia; june_lee@moh.gov.my

**Keywords:** microalgae, industry, isolation, bioactive compounds, nutraceuticals, pharmaceutical, cosmeceutical

## Abstract

Microalgae are known as a rich source of bioactive compounds which exhibit different biological activities. Increased demand for sustainable biomass for production of important bioactive components with various potential especially therapeutic applications has resulted in noticeable interest in algae. Utilisation of microalgae in multiple scopes has been growing in various industries ranging from harnessing renewable energy to exploitation of high-value products. The focuses of this review are on production and the use of value-added components obtained from microalgae with current and potential application in the pharmaceutical, nutraceutical, cosmeceutical, energy and agri-food industries, as well as for bioremediation. Moreover, this work discusses the advantage, potential new beneficial strains, applications, limitations, research gaps and future prospect of microalgae in industry.

## 1. Introduction

**Figure 1:**
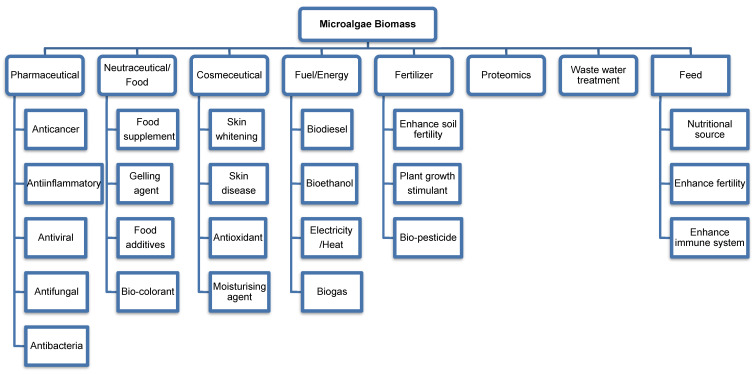
Potential uses of microalgae in various industries.

**Figure 2:**
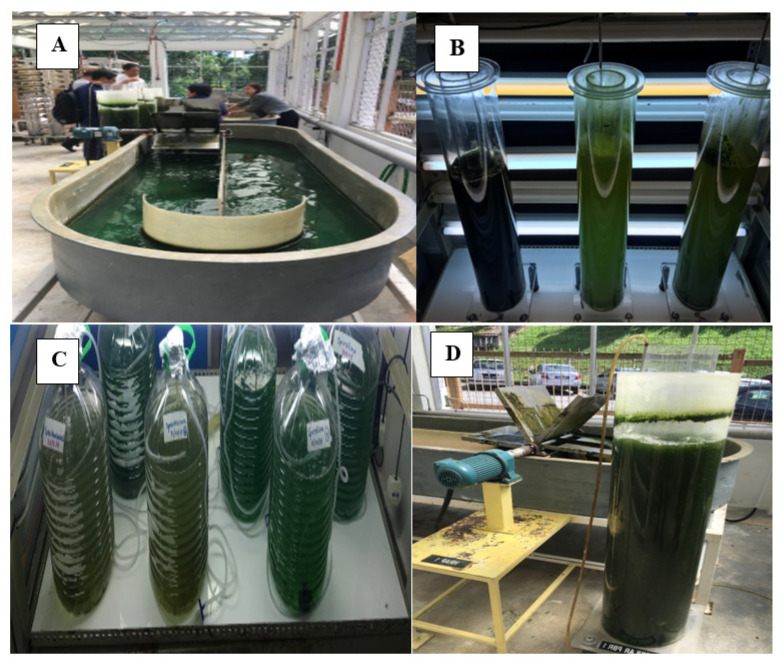
Lab scale microalgae cultivation types. (**A**) raceway photobioreactor; (**B**) indoor annular column photobioreactor; (**C**) microalgae culture in bottles with light and oxygen in Scientific laboratory; (**D**) open annular photobioreactor (Courtesy: Dr. Adibi Rahiman Md Nor, University Malaya).

Microalgae are in the form of unicellular, multicellular, filamentous or siphonaceous, known as photosynthetic microorganisms that can be categorized as eukaryotic and prokaryotic [1]. Microalgae are also the largest global primary producers that consist of approximately 200,000 species [2] with distinctive nutrient contents as well as bioactive compounds which have a wide spectrum of commercial applications in various facets of industries including pharmaceuticals, nutraceuticals, cosmeceuticals, biofuels, biofertilisers, wastewater treatments, feed, and proteomics (Figure 1). Production of microalgae involves mass cultivation, recovery of biomass and downstream processes for sustainable yield to cater for food, chemical, feed, biofuel, and high value products. Intrinsic factors such as temperature, salinity, light, and the availability of nutrients affect the chemical composition of the biomasses. Figure 2 illustrates a few of the cultivation processes of microalgae.

The applications of microalgae in industries are concentrated in a few specific species that has high economic value. The highly-sought genera in the global algae market were dominated by Spirulina and Chlorella in the form of dried biomass due to various beneficial health effects [3]. Among the myriad components which were exploited for commercial purpose are fatty acids, carotenoids, vitamin, minerals, polysaccharides, and bioactive compounds. According to the latest analysis, the global market for microalgae is forecasted to reach USD 3318 million by 2022 driven mainly by the demand from pharmaceutical and nutraceutical industries [4] owing to customers’ increasing health concern, interest on natural alternatives as well as escalating chronic diseases. Likewise, various microalgae derived compounds were reported to exert various skin benefits which are currently gaining attention in many aspects of cosmeceuticals. Advances of new application areas of microalgae in aquaculture and biofuel production have provided a significant rise of algae demand in the global market. Microalgae are constituted of a high level of lipids. Various research has highlighted the potential of microalgae biomass as a source of renewable energy, namely biofuel which is imperative to reduce the dependency on fossil fuel [5]. Microalgae have an upper hand in the biofuel production compared to other bioenergy sources (corn, sugar cane, palm oil, etc.) as they do not require arable land for cultivation, thus eliminating competition for space and resources with food crops. Some of the key players in the algae industries were Algae Tec, Pond Biofuels Incorporated, Cyanotech, Kai BioEnergy, Algae Systems and others [4].

The review aims to summarise the value-added components from microalgae with potential application in the pharmaceutical, nutraceutical, cosmeceutical, energy and agri-food industries, as well as for bioremediation along with commercial applications and examples of microalgal manufacturers as well as commercialized products.

## 2. Microalgae Biomass

The mass production of algae biomass is important for various industries [6]. Numerous methods have been established for microalgae-based products development and down-stream processes with the advancement in the technologies in this area.

### 2.1. Biomass Production

**Figure 3:**
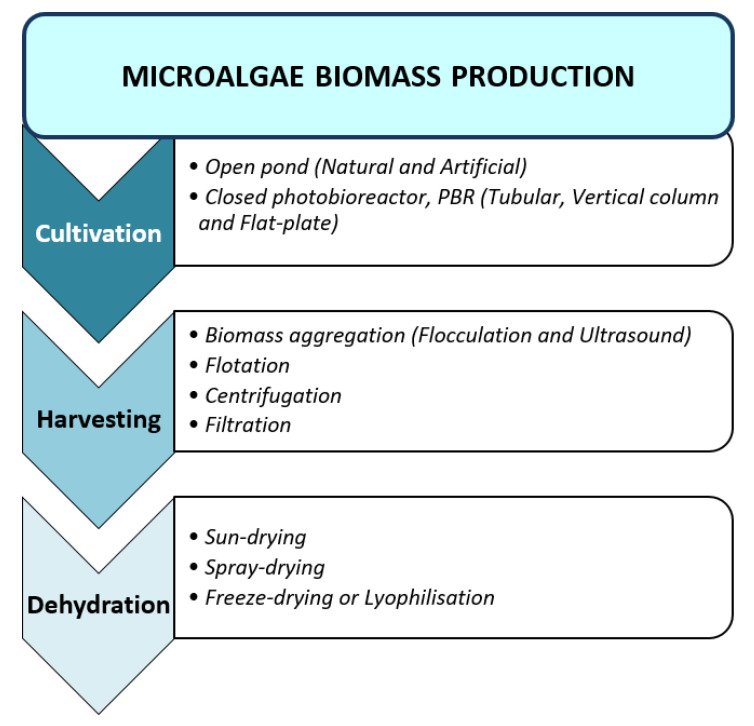
The production of microalgae biomass [5,6].

The process of biomass production of microalgae encloses several steps such as cultivation, harvesting and biomass dehydration as shown in Figure 3.

#### 2.1.1. Cultivation

There are two systems developed for the production or culturing of algal biomass: the open pond and closed photobioreactor (PBR) technologies. Open pond production is categorised into two systems: natural waters (ponds, lakes and lagoons) [7] and artificial ponds (circular and raceway) [7,8]. The open pond is a cheaper method of large-scale algal biomass production compared to the PBR. The PBR, however, provide an excellent and controlled closed culture system for cultivation, preventing hazard or contamination from moulds, bacteria, protozoa and competition by other microalgae [9]. It is usually placed outdoors to exploit the free sources of energy from sunlight. There are three types of PBR categorised into tubular (TPBR), vertical column (VCPBR) and flat-plate (FP-PBR) [10].

#### 2.1.2. Harvesting

The microalga biomass can be separated from the culture medium or harvested by four means: biomass aggregation (flocculation and ultrasound), flotation, centrifugation and filtration. In some cases, combinations of two or more techniques are used to increase effectiveness. The harvesting method selection depends on several criteria of the microalgae such as the density, size and the desired final products [11].

(a) Biomass aggregation: In the flocculation technique, microalgae cells are aggregated together to form a larger particle known as floc, with the addition of flocculants such as multivalent cations and cationic polymers to the media which helped to neutralise the cells surface charge [12]. There are two types of flocculating agents: chemical and bio-flocculants. The cheaper and easily available chemical flocculants that are widely used in industry are iron and aluminium salts [13]. Meanwhile, the common biopolymer bio-flocculants used include acrylic acid and chitosan [14]. In the ultrasound technique, aggregation is initiated followed by increased sedimentation to facilitate harvesting of the algal biomass [15]. The benefit of using this method is that the valuable metabolites are preserved because ultrasonic harvesting does not produce shear stress on the biomass although used continuously [16].

(b) Flotation: It is a technique intended to float algal cells on the surface of the water, using a micro-air bubbles disperser without the addition of chemicals [17]. This technique is economically advantageous due to the low operational costs with an easy operating procedure and high harvesting of biomass [18].

(c) Centrifugation: It is the recovery of algal biomass from the culture media using a centrifuge by gravitational force [19]. This technique is rapid, easy and efficient, but the cost can escalate due to the high energy input and maintenance required [20]. Another disadvantage of this technique is the internal damage of cells, causing a loss of delicate nutrients if a high gravitational force is used [21].

(d) Filtration: It is a process to isolate alga biomass from the liquid culture medium by using a porous membrane with various particle size ranges [22]. It can be implemented through three different ways: conventional, microfiltration and ultrafiltration (isolation of metabolites). The conventional filtration is used to harvest large size microalgae (>70 μm) such as *Coelastrum* and *Spirulina*. Microfiltration and ultrafiltration are used to harvest smaller size microalgae, equal to the size of the bacteria [23].

#### 2.1.3. Biomass Dehydration

Algae biomass is immediately processed to the following stage after being separated from the culture medium to prevent spoilage or to extend their shelf-life [22]. Three different types of drying or dehydration process that are normally used include sun-drying, spray-drying and freeze-drying. The method chosen is entirely dependent on the desired final products:

(a) Sun-drying: It is the cheapest method available compared to the other two techniques. This technique is solely based on the solar energy which causes limitations in terms of weather condition, long drying period and the large drying area needed [24]. Since drying using sunlight is an uncontrollable process, the problem of overheating may occur, change of texture, colour and taste of the microalgae [22].

(b) Spray-drying: This technique is to produce dry powder from a fine spray of suspension droplets which is in continuous contact with hot air in a large vessel. This method has many advantages such as can be operated continuously, the powder produced is very fine and the rapid drying can maintain a good quality product [25,26]. This method is usually opted for high-value operations due to its efficiency, but some algal components such as pigments can be significantly deteriorate and the operation cost is expensive [22].

(c) Freeze-drying or Lyophilisation: It is widely used at laboratory-scale only to dry microalgae since large scale production can be very expensive [20]. Freeze-drying is a direct dehydration process of frozen products using sublimation mechanism. The microalgae are frozen to solidify the material within before freeze-drying. The moisture content of the microalgae is decreased slowly at low-temperature, maintaining the solid structure and the quality of the product [27].

### 2.2. Extraction of Bioactive Compound

The microalgae are composed of carbohydrates, lipids, proteins, minerals and many other compounds. To utilise the compounds for different application such as for biofuels/energy and agricultural use, the algal biomass will be pre-treated to release the stored bioactive compound in the cells [28]. The cell walls will be lysed to enable all the desired components to be extracted, and this may be accomplished by various ways, such as physical, mechanical (bead milling, homogenisation, microwave, ultrasonic and pulsed electric field), chemical (solvent, acid and alkali) and biological (enzymes) methods. The method chosen for the pre-treatment process is based on the desired final products [29,30].

## 3. Microalgae in Pharmaceuticals

Microalgae are a potential source for bioactive components with pharmaceutical applications. Several important microalgae-derived components with their pharmaceutical applications are highlighted in Table 1.

Many studies have documented the health benefits of microalgae compounds for prevention and improvement of diseases such as diabetes, obesity, cardiovascular disease, cancer, inflammation, Alzheimer’s diseases, depression as well as bacterial, fungal, and viral infections [38,48,49]. Despite this, only a limited number of microalgae with pharmaceutical applications are currently available as listed in Table 2. Low extraction yield and high production cost are some of the factors in delaying commercialization of some microalgae-derived bioactives [50]. In addition, there is a potential risk of severe side effects, allergic reactions, and accumulation of heavy metals and toxins in some species of microalgae [51]. Consequently, strong emphasis on the good manufacturing practices in cultivation, harvesting, extracting and purification and controls to limit toxin and impurities are required to ensure safety, efficacy, and quality of microalgae-derived purified compounds and enriched extracts for approval and commercialization [52].

### 3.1. Compounds with Anti-Cancer Properties

Microalgae-derived bioactives with anti-cancer properties are extensively studied in recent years [52]. Among the compounds, the microalgae pigments such as astaxanthin, β-carotene, lutein, violaxanthin, and fucoxanthin have the most potential to be commercialized as pharmaceuticals because of their established applications as nutraceuticals and cosmetics and the rising demand as dietary supplements. In fact, astaxanthin and β-carotene are currently produced commercially from microalgae *Dunaliella salina* (*D. salina*) and *Hamatococcus pluvialis*, respectively, while commercial production of other carotenoids such as lutein and fucoxanthin are gaining momentum [56]. Fucoxanthin, for instance, have been isolated from diatom microalgae *Phaeodactylum tricornutum*, which is cultivated in a pilot-scale photobioreactor and can be considered as a commercially viable source for fucoxanthin [48]. The anti-cancer properties of some of these carotenoids are summarized in Table 1. Extraction of carotenoid is achieved using ultra-sound extraction and freeze-thawing methods; however, organic solvent extraction at high temperature and pressure is a more widely used method in commercial-scale production [56].

Another promising anticancer agent is phycocyanin, a protein pigment from the phycobiliprotein group. Phycocyanins are isolated from the commercially grown microalgae *Spirulina platensis*, but isolation from other cyanobacteria such as *Limnothrix* sp. has also been reported (Table 1) [38,40]. C-phycocyanin showed inhibitory activity in liver cancer cell lines (HepG2) [34], human leukemia cells (K562) [35] and against lung cancer cell lines (A549 and NSCLC) [36,37,38]. In another study, the phycocyanin from *Limnothrix* sp. enhanced the anticancer properties of the anticancer drug Topetecan against the prostate cancer cell line (LNCap) [39]. Meanwhile, phycocyanin isolated from as *Limnothrix* sp. NS01 with two subunits α and β (17 and 20 kDa) showed antiproliferative activity in human breast cancer cell lines (MCF-7) [40]. Isolation of C-phycocyanin from *Spirulina platensis* was accomplished using buffer ammonium sulfate solution (i.e., salting-out technique) as well as by using supercritical fluid extraction using ethanol as a modifier which resulted in higher yield compared to conventional solvent extraction [34,38]. In the studies, impurities were removed using chitosan and activated charcoal however further purified the compound by ion-exchange chromatography was also carried out [40].

### 3.2. Compounds with Cardioprotective Properties

Several microalgae-derived compounds have also been studied for its cardioprotective effect and briefly summarized in Table 1. Carotenoids are shown to possess antioxidant properties that are important for preventing cell damage caused by free radicals associated with chronic cardiovascular diseases and stroke [57]. In this regard, some microalgae producing a high amount of carotenoids have been investigated for their cardioprotective properties. For instance, *D. salina* microalgae can produce up to 10–13% of β-carotene have been shown to have protective effects against atherosclerosis in both mice and humans. Furthermore, a mixture of *tran*-isomers (~40%) and *cis* β-carotene isomers (~60%) from *D. salina* was found to be more potent in decreasing total lipid, cholesterol and triglyceride (TG) levels compared to all *trans*-β-carotene found in synthetic β-carotene [58]. Harari et al. [41] also showed that *cis* β-carotene isomers (~50%) from *Dunaliella bardawil* powder inhibited atherosclerosis progression in older mice with a high-fat diet. Although *cis* β-carotene is still not produced commercially due to its high production costs, a high density inoculum enriched with *cis* β-carotene strain from *D. salina* are currently being developed to deliver reproducible, low-cost *D. salina* biomass containing a high content of 9-*cis* β-carotene [50].

Another group of bioactive with cardioprotective properties are the polyunsaturated fatty acids (PUFAs), especially the omega-3 fatty acids such as DHA, EPA and α-linoleic acid (ALA) which have been shown to reduce blood cholesterol and improve hypertension. Of these fatty acids, DHA is the only PUFA currently commercially available. Purified EPA sourced from various microalgae including *Porphyridium purpureum* and *Isochrysis galbana* are still not economically competitive to be produced commercially. However, a proprietary strain of microalgae *Nannochloropsis* cultivated in an open pond with high solar radiation was shown to produce high EPA content (>65%) oil marketed as A2 EPA Pure^™^ for supplement and pharmaceutical applications have been reported [59]. PUFAs are commercially extracted using a hexane solvent followed by mechanical pressing. However, extracted PUFAs are prone to oxidation and therefore all materials that can initiate oxidation such as copper are eliminated from the extraction and storage area [59].

### 3.3. Compounds with Antiviral Properties

Microalgae are also potential sources for bioactives with antiviral properties (Table 2). For example, lectin protein with antiviral properties such as cyanovirin (CV-N) and scytovirin (SVN) have been reported [43,44]. CV-N is a 11kDa protein consisting of 101-amino acid in single chain with two disulfide linkages and was isolated from the aqueous extract of *Nostoc ellpsosporum* and purified by ethanol precipitation followed by fractionation and purification by column chromatography [60]. CV-N showed a broad spectrum of antiviral activity against human immunodeficiency virus-1 (HIV type-1) laboratory and clinical strains with effective concentrations (EC_50_) range 0.1–5.8 nM and 1.5–36.8 nM, respectively [43]. In the same study, CV-N was also active against human immunodeficiency virus-2 (HIV-2) and simian immunodeficiency virus with EC_50_ 2.3–7.6 nM and 11 nM, respectively [43]. Although CV-N showed promising antiviral activity including in an animal HIV transmission model the clinical application of this molecule is currently limited due to its reported mitogenic activity [61]. Meanwhile, another lectin protein SVN has been isolated from the aqueous extracts of cultured cyanobacterium *Scytonema varium*. SVN is a 9.71kDa protein consisting of 95-amino acid chains with 5-disulfide linkage which also showed potent antiviral activity against HIV type-1 laboratory strains and clinical isolates with EC_50_ between 0.3–22 nM [44].

Another group of compounds with antiviral activities are the cyclic peptides. The fractions containing a mixture of Icthypeptins A and Icthypeptins B, cyclic depsipeptides isolated from *Microcystic ichthyoblabe* showed antiviral activity against influenza A virus with inhibitory concentration (IC_50_) of 12.5 µg/mL comparable to the control amantadine IC_50_ of 15 µg/mL [45]. Besides that, sulfated polysaccharides with antiviral properties have also been described. For example, calcium spirulan isolated from *Spirulina platensis* showed antiviral activity against HIV-1 with IC_50_ of 9.3 µg/mL comparable to the dextran sulfate control when assessed by the P24 antigen assay [46]. The sulfated exopolysaccharide (1 µg/mL) from the Spanish strain of *Porphyridium cruentum* showed a strong inhibitory effect on the cytopathic effect on Herpes simplex virus-1 (HSV-1), Herpes simplex virus-2 (HSV-2) and Vericella virus (VZV) with CPE_50_ protection of 0.7–5 µg/mL [47].

## 4. Microalgae in Nutraceuticals/Food

A number of microalgae have been classified as Generally Regarded as Safe (GRAS) and approved by the US Food and Drug Administration (FDA). For the guaranteed safety and valuable source of nutrients, algae are used widely in industries especially for food and nutraceutical applications. According to Watanabe [62], microalgae species of cyanobacteria eg. *Spirulina*, *Aphanizomenon* and *Nostoc* are hugely harvested for the food industry. Dried *Aphanizomenon* contributes approximately 500 tons annually, which is dominantly produced in North America at Upper Klamath Lake, Klamath Falls, Oregon for the food supplement industry, while *Spirulina* is widely cultured and produced in countries like United States, Taiwan, China, India, and others with an estimated output of 3000 tons annually [63]. The Myanmar Spirulina factory in Yangon produces tablets, chips, pasta, and liquid extract [3]. Other species like *Chlorella* sp., *Haematococcus* sp., and *Dunaliella* sp. cultivated commercially by various countries for nutraceuticals and food application. The list of commercialized strain, industry application, companies, and the countries involved are provided in Table 3.

### 4.1. Algal Protein

Demand for plant-based nutrient especially protein sources has augmented over the years owing to growing health concerns and a shift of millennial preference across the world to nutraceutical products which are convenient and offers a high value of nutrition. This, in turn, has the manufacturers and industries of food and beverages as well as nutraceuticals to search for lucrative sources of protein. Among all, microalgae showed as a promising source of protein combined with diverse bioactive compounds and essential nutrients. The demand for global algae protein exceeded USD 700 million in 2019 and projected to expand over the years in view of changing lifestyle of consumers and preference [78].

Among the myriad of algae, *Chlorella* and *Spirulina* species are most sought-after in the global microalgae market owing to its high protein content (50–70% protein of its dry weight) and broad spectrum of other nutrients viz. minerals, vitamins, lipids, carbohydrates, pigments and other trace elements [79]. Tavelmout Corp., a biotech company based in Japan developed a closed flat panel photobioreactor system which enhances protein productivity in *Spirulina* about 20 times higher than that of soybeans [80]. While, Siva Kiran et al. [81] reported that the protein content in *Spirulina* is higher compared to other foods such as milk, chicken, beef, and some plants. In food application, *Spirulina* and *Chlorella* or its protein were incorporated in various types of food, such as milk-based products, bread, biscuits, instant noodles and pasta to produce protein-enriched functional food [82,83,84,85].

The extraction of protein from microalgae comprised steps including cell disruption, extraction and product purification; cell disruption techniques involve mechanical action (high-pressure homogenisers, bead mills), ultrasounds, enzymatic or chemical treatments, thermal or osmotic shocks (repeated freezing/thawing) [86]. For efficient protein recovery (76%) from *Chlorella* sp., Ursu et al. [87] suggested an alkaline treatment followed by isoelectric precipitation. Meanwhile, Chia et al. [88] proposed an effective approach using ultrasound-assisted three phase partitioning method for efficient protein extraction and an optimised conditions for high protein recovery which is applicable for the future integrated bio-separation technique for biomolecules extraction from microalgae as well as to improve the current downstream bioprocessing techniques.

*Chlorella* and *Spirulina* are known for its high-quality protein attributed by the well-balanced amino acid composition according to FAO/WHO recommendation [89], digestibility coefficient, as well as by its biological value of the amino acids absorbed from the food [90]. Nevertheless, interest to acquire good quality protein for human nutrition continues with exploration on different strains of microalgae, as such, a study on Australian microalgae species in James Cook University/MBD Energy Research facility using *Scenedesmus* sp., *Nannochloropsis* sp., *Dunaliella* sp., and a designed freshwater chlorophytic polyculture (CPC; consisting of *Schroederiella apiculata, Scenedesmus pectinatus, Tetraedrom minimum, Mesotaenium* sp. and *Desmodesmus* sp.) exhibited high quality protein in all studied microalgae. The protein was suitable for human consumption which was determined and supported by Essential amino acid index (EAAI) and was comparable to the commercial *Spirulina* and *Chlorella* products. The Australian strains earned higher score for EAAI due to presence of higher essential amino acids such as histidine, phenylalanine, threonine and lysine. In addition, the selected strains also displayed a comparable nutrient strength and taste as the *Spirulina* and *Chlorella* species respectively, thus, suggesting the potential of these microalgae for future commercialization in human nutrition area [91].

### 4.2. Vitamins and Minerals

Microalgae constitute important source of almost all vitamin and essential minerals. *Spirulina* was reported as rich source of vitamins B_1_, B_2_, B_12_ and high content of amino acid up to 62%, [92], all of which have facilitated its claim as superior to other microalgae [93]. Seghiri et al. [92] also suggested that *Spirulina* benefits may be attributed by the presence of macro-minerals as well as the trace elements. Various studies have shown that *Spirulina* as a good source of pro-Vitamin A, in term of beta-carotene and exhibited better effects than the synthetic Vitamin A due to its good bioavailability [94,95]. This species also was reported for high content of Vitamin B_12_, in the range of 120–244 µg per g dry weight, however, studies suggested that most of it were pseudo B_12_, an analogue which has a similar structure and bound to specific B_12_ transporter but does not exert any health benefit [62,96]. In contrast, the latest study by Madhubalaji et al. [97], provided a scientific validation for the use of *Spirulina* as Vitamin B_12_ source. However, more studies needed to substantiate *Spirulina* as a viable source of Vitamin B_12_ in human. In view of high bioavailability of iron from *Spirulina*, Puyfoulhoux et al. [98] concluded that Spirulina constitute adequate source of iron for human consumption.

*Chlorella* is another commercial species with a rich source of proteins, essential amino acids, vitamins (B-complex, ascorbic acid), minerals (potassium, sodium, magnesium, iron, and calcium). *Chlorella* sp. is suggested to be the best candidate for vegan source of Vitamin B_12_ and used in food supplement [99]. According to Kumudha et al. [66], Vitamin B_12_ detected in the *Chlorella vulgaris* present as methylcobalamin, a biologically active form suitable for human consumption while Merchant et al. [100] reported that participant supplemented with 9 g *Chlorella pyrenoidosa* daily mitigated vitamin B_12_ deficiency in vegetarian and vegan participants. A study by Nakano et al. [101], suggested 6 g *Chlorella* supplementation significantly reduced the risk of pregnancy-associated anaemia, proteinuria and oedema. All of these, suggest microalgae enriched food as a good source of nutritional supplements especially for strict vegetarians owing to its rich high-value nutrients as well as Vitamin B_12_. Chronopoulou et al. [102] proposed an improved approach to extract fat-soluble vitamins by using supercritical CO_2_ while a previous study compared six extraction methods for Vitamin B_12_ from Spirulina and found the aqueous extraction suits best for this studied compound [103].

### 4.3. Fatty Acids

Microalgae contain distinctive profile of lipids especially the fatty acids, for example, EPA or DHA with feasible commercial value. The ability to produce and accumulate high amount of PUFAs, makes microalgae even more valuable as nutraceutical. Omega-3 fatty acids known as a good source of dietary supplements which highly recognised and recommended for its health benefits specifically in disease prevention [104] and human nutrition. The algal oil often used in liquid or capsule form and benefits in particular, vegetarians as well as populations with low seafood diets. Application of algal oils in food achieved popularity after advancement in the microencapsulation and refining technology which prevents any off-flavour towards the food when combined together with the oils. The algal oils are enriched or fortified in myriad of foods such as dairy products, nutritional bars, bakery products and etc. [105] to further increase product nutritional value.

Microalgae produce total lipid up to 30–70% of dry weight depending on the species; which plays a critical role as energy stockpile during adverse condition and cell division. Fatty acids are component of complex lipids and the structural variation attributes to their multitudinous benefits. Based on the polarity of the molecular head, fatty acids can be categorised into two groups; (i) neutral lipids and (ii) polar lipids. Fatty acids production can be increased in the microalgae by manipulating various environmental factors such as oxygen level, temperature, light exposure, pH as well as limiting the nutrient supplementation [106]. A number of studies have suggested nitrogen starvation to increase the neutral lipid and triacylglycerides (TAG) synthesis in microalgae [107,108]. Meanwhile, exposure to low light intensity and therefore low temperature was reported to enhance PUFAs production [109,110]. *Chlorella* sp. culture in low CO_2_ showed to promote high contents alpha-linolenate fatty acid, in contrast *Chlamydomonas reinhardtii* mutant cia-3 exhibited higher content of PUFAs in culture condition with high CO_2_ concentration [111,112].

Methods of harvesting lipid from microalgae include mechanical pressing, homogenization, milling and solvent extraction. The solvent extraction is imperative to the polarity or/and solubility of the lipid of interest. Other techniques such as enzymatic extraction, supercritical extraction, ultrasonic-assisted extraction, microwaves are used to facilitate lipid extraction by solvent [113]. Nevertheless, only a few cell disruption techniques feasible for commercial application, namely, steam explosion [114], enzymatic hydrolysis, bead milling and horn sonication [113]. Purification of algal oil (PUFAs) is an essential step as the crude form of these oils are inedible due to its impurities, odour, taste and unattractive for consumption as its turbid appearance. Speed of operation and process condition are critical factors in the oil purification techniques as the oil is sensitive to oxidation. Manufacturer such as Martek Bioscience Corporation has described the oil recovery and purification steps of DHA from algae oil. For instance, protease enzyme was used to break the protein in the cells of *Schizochytrium* sp. to release the oil into the culture broth forming an emulsion, thereafter isoprophyl alcohol was added to separate the oil. While for *Crypthecodinium cohnii*, hexane solvent extraction method was applied since the enzyme method is incapable to hydrolyse the algae cellulosic layer. Following the solvent extraction, the cell walls are removed by centrifugation and the oil was recovered after solvent evaporation [115].

Several studies and reviews have been published on the different algae strains, namely, *Nannochloropis oculata* [73,74], *C. cohnii*, *Schizochytrium* sp., *Ulkenia* sp. which are associated with the presence of high concentration of PUFAs in its lipid [116] and manifested to have better bioavailability compared to other fish oils [74]. These strains have been commercially used to develop high purity marine oils by companies such as Qualitas Health, DSM-NP, Lonza and GCI Nutrients. DHA oil from *C. cohnii* (40–50%) are fortified in infant formula milk by company such as Martek, USA, thus, the cultivation and manufacturing processes follow a strict regulation of FDA and current Good Manufacturing Practice (cGMP). This fortified formula sold in more than 60 countries [3]. Recently, two more strains with potential commercial values namely, *Phaeodactylum tricornutum* and *Porphyridium purpureum* for EPA and other compounds were suggested [117,118].

### 4.4. Natural Pigments

**Figure 4:**
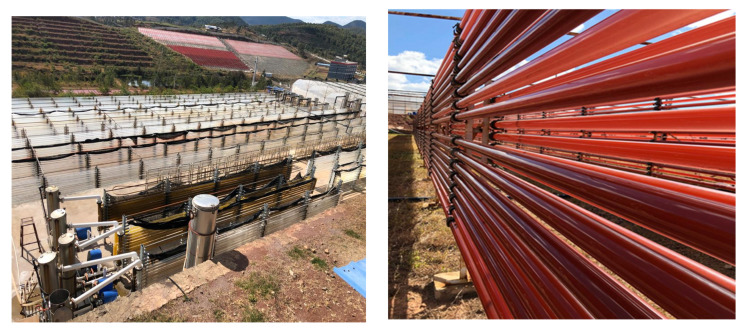
Closed tube bioreactor algae production facility in Yunnan Province, China (Courtesy: Nicholas Cheong, Nova Laboratories Sdn. Bhd.).

Microalgae are also a rich source of pigments which are used as bio-colourant or food additives in various products. Natural pigments are highly coveted by food industries as an alternative to synthetic sources which has various health implication [119]. One of the key players in this natural pigment industry is a Chinese astaxanthin supplier, Algae Health Science who operates one of the biggest facilities in the world, using glass tubes to cultivate *Haematococcus pluvialis* microalgae for astaxanthin extraction in Yunnan, China (Figure 4) [120].

Closed system cultivation was favoured by this company as it provides better purity and prevents contamination, independent of local weather as well as providing consistent production. Astaxanthin from *Haematococcus* has been marketed under various name and companies, as such Cyanotech Corp. from the US commercialized astaxanthin under the name of BioAstin [121], AstaReal by Fuji Chemical Industry, Japan, Astaxanthin Gold^™^ by Nutrigold as well as various patent applications for dietary supplements, health supplements, as antioxidanst, beverage colourants and other functions [122,123].

Chlorophylls, a photosynthetic green pigment present in microalgae as chlorophyll *a* (blue-green colour), *b* (brilliant green), *c* (yellow-green), *d* (brilliant/forest green) and f (emerald green) dependent on types of algae [124]. Chlorophyll *a*, which is abundantly found in cyanobacteria such as *Chlorella*, has been extensively used as colouring agent due to its stability [125]. Another prominent colourant from the microalgae with commercial applications is phycobiliproteins, a water-soluble fluorescent pigment commonly present in cyanobacteria which can be classified as four major groups according to their colours and light absorption characteristics, namely, phycoerythrin, phycocyanin, allophycocyanin and phycoerythrocyanin [126,127]. The phycobiliproteins were shown as a strong antioxidant which contributes to high-value nutraceutical product and its application in food mainly in dairy products, chewing gums, candy, beverage mixes and ready-to-eat cereals [128,129]. Besides being a functional ingredient and safe food colourant, this protein (eg. deep blue colour protein C-phycocyanin) are used as dietary supplement coating and functional food additives [85].

Since the global demand of natural colourant especially astaxanthin is growing at an unprecedented rate mainly for food supplements, thus, their average prices in the market are in the range of 2,500 USD per kilograms and its annual worldwide worth of 200 million USD [130]. Species like *Dunaliella* ascribed to its high beta-carotene contents, contributes a total turnover of about 75 million USD where 60 million from it contributed by food supplements [64].

Many reviews and research papers offer detailed steps on how to improve and enhance related algae cultivation and extraction methods. Development of spiral-tube photobioreactor to cultivate *D. salina* contributes to an enhanced and continuous cultivation for high output of β-carotene [131]. Additionally, factors such as high light intensity, temperatures, nutrient limitation and high salt concentrations increase the β-carotene production [132]. A recent study by Xu and Harvey [133], suggested the use of high-intensity red light with sufficient nutrient content for high carotenoid production by up-regulating the whole biosynthesis pathway of carotenoids. Besides its applications as a food colourant, beta-carotene is used as an additive in multivitamin supplement and tablets [3].

Harvesting and extraction of certain algal strain can be challenging due to its nature, as such *D. salina* harvesting deemed to be most difficult and costly compared to other commercial strains attributed to its lacking of cell walls, low concentration and small size. Many approved techniques have been developed to tackle the problem such as centrifugation and flocculation, a patented method involving mechanical harvesting [134] while Pirwitz et al. [135] suggested centrifugation without flocculation method which was identified as most cost-effective technique. Other commonly used extraction methods for pigments such as astaxanthin are by mechanical treatments, chemical treatments, pressurized extraction, ultrasound and microwaves among others while a few researches have taken a greener option by using non or less toxic solvents such as acetone and ethanol for the extraction process [68]. Molino et al. [67] suggested a mechanical pre-treatment before accelerated extraction using green solvents.

A continuous search for potential new strains for large-scale production of these pigments are still advancing as the current major strains are still limited to a few species, namely, *Spirulina platensis, Haematococcus pluvialis* and *Chlorella* sp. Singh et al. [136] discovered *Asterarcys quadricellulare* PUMCC 5.1.1 strain, a green microalga with a promising characteristic for carotenoid production while Kaushal et al. [137] reported a new cyanobacterium *Nodularia sphaerocarpa* PUPCCC 420.1 which produces phycobiliprotein and reckoned as a good candidate for production at commercial scale.

## 5. Microalgae in Cosmeceuticals

Skin entity is the largest organ which acts as a physical barrier to protect the human body from harmful external agents, and is composed of three main layers (epidermis, dermis, hypodermis) [138,139,140]. A healthy and radiant skin is maintained via a balance between synthesis and degradation of the matrix proteins such as collagen, elastin and glycosaminoglycans (hyaluronic acid). The corresponding proteinases coupled with continual synthesis are the maintenance mechanism involved in this process. However, this balance is affected via both chronological as well as photo-ageing by coupling the proteinases up-regulation, where this enhances protein degradation along with synthesis down-regulation. Protein synthesis simulation or alteration of their breakdown via proteinases is the suggested mechanism of action for improving this inequality [141].

The synthesis of matrix proteins or proteinases inhibition are attempted with the application of a wide range of specific skin formulation products containing compounds such as ethanolamines, sodium lauryl sulphates, polypeptides or oligopeptides. However, the possibility of developing allergic reactions and other adverse conditions led to continuous search for natural bioactive compounds that could be applied in cosmetic formulations. As such, bioactive compounds originating from microalgae have gained much popularity of its amazing moisturizing, thickening, pigmenting, anti-ageing, skin whitening, sunscreen protection, and many other properties [140,141].

**Figure 5:**
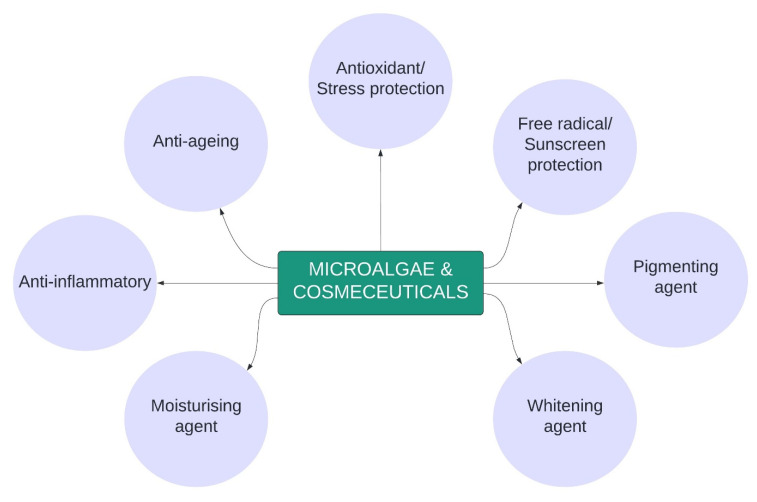
Potential applications of microalgae in cosmeceuticals.

In a review by Mourelle and colleagues, cosmetics are defined as products aimed for improving skin appearance, structure and morphology in assistance of excipients and active ingredients specifically suited for various skin types [142]. Several microalgae species extracts are widely applied in cosmetic-based industries, especially for skincare products. These include face and skincare (anti-ageing cream, emollient, anti-irritant in peelers, refreshing care, sunscreens) as well as hair care products [143]. Among the common microalgae species include *Arthrospira sp., C. vulgaris, D. salina, S. platensis, Chondrus crispus, Mastocarpus stellatus, Ascophyllum nodosum, Alaria esculenta* and *N. oculate* [144]. In cosmetic industry, Cu-Chl (CI 75810) is applied for use in hair colour products, colour cosmetics and bleaching products and categorised as non-toxic [140]. Figure 5 highlights the main potential applications of various microalgae in cosmetic industry, accounted by its metabolites or bioactive compounds, which are described in detail below.

### 5.1. Anti-Ageing

Ageing is a process that involves a decrease in skin structural proteins (elastin, collagen, hyaluronic acid) synthesis leading to loss in skin elasticity, laxity, integrity and finally gives rise to visible signs of ageing. Apart from decreased protein synthesis, age-dependent down-regulation of skin elasticity is also contributed by an up-regulation of the corresponding proteinases. Hence, the most appropriate anti-ageing strategy in order to attain smooth and healthy skin is by controlling the skin structural constituents’ degradation via proteinase activity regulation. In addition, frequent and repetitive exposure to ultraviolet (UV) results in over-production of reactive oxygen species (ROS) that induces oxidative cellular stress and promotes genetic alterations thereby affecting matrix protein structure as well as functions, which eventually results in skin damage. In order to overcome this issue, there is a strong need to scavenge these free radicals [141].

β-carotene, is one of the microalgae pigments commonly synthesized by *D. salina* that assists in the prevention of free radicals that initiate premature ageing [140,145,146]. Another carotenoid compound, lutein is also reported for its protective reactions towards UV radiation and can be obtained from several microalgae (*Scenedesmus salina, Chlorella, C. vulgaris, Scenedesmus obliquss, D. salina* and *Mougeotia* sp.) [140,147]. Lycopene is also a microalgae carotenoid pigment that is employed in skincare products for its anti-ageing properties where this compound neutralizes oxygen-derived free radicals [140,142,148].

### 5.2. Antioxidant/Stress Protection

Oxidative stress process impacts the skin mainly in terms of premature ageing, uneven skin tone/texture and also might disintegrate the essential proteins. Once the collagen and elastin in the dermal skin layer are diminished via oxidative stress, this also involves significant DNA damage, inflammatory response, reduced antioxidant protection and the generation of matrix metalloprotein. In the long run, this results in expedited ageing process along with significant appearance of wrinkles with loss of elasticity [149]. Fucoxanthin, that is predominantly present in *Phaeodactylum tricornotum, Odontella aurita* and *Isochrysis aff. Galbana* is one important metabolite that was identified to portray antioxidant activity as well as preventing oxidative stress. Astaxanthin is reported to possess higher antioxidant activity compared to vitamin A or E, and a recent study by Davinelli and co-workers in the year 2018 highlighted its potential as a potent anti-wrinkle and antioxidant agent [150].

### 5.3. Free Radical/Sunscreen Protection

UV radiation is reported to be beneficial in limited duration; however, prolonged exposure is not advisable as this will result in severe skin damage. In order to prevent these harmful effects, various skincare products are usually applied especially by women that could range from sunscreens, sunblock lotions or anti-ageing serum. Microalgae-derived carotenoid pigments such as astaxanthin, lutein, zeaxanthin and canthaxanthin found abundantly within *Dunaliella* and *Haemotococcus* sp. are noted for their protective properties against extensive sun damages [151]. In addition, orange-pigmented violaxanthin compound isolated from *Nannochloropsis oceania* is proven to significantly block UVB-detrimental effects along with decreased cell viability and increased ROS production [152]. Fucoxanthin, is another microalgae-derived pigment that has been shown to impart protective effect against sunburn [153].

### 5.4. Pigmenting Agent

Almost all cosmetic products are incorporated with synthetic colourants which attracts much attention of the cosmetic industry towards identifying colourants from natural sources for long-term sustainability. Microalgae pigments fit well into these criteria where it is classified as a naturally sustainable source with added health benefits that include UV protection, anti-ageing, antioxidant and anti-bacterial properties. Carotenoids, chlorophylls and phycobiliproteins encompass the major classes of pigments in microalgae; where they impart various colours ranging from green, yellow, brown and red making it suitable as an alternative to synthetic colourants [154]. Chlorophylls are extensively applied for use as cosmetic colourants, along with its other use in deodorants as an odour-masking agent. Chl a, predominantly found in *Chlorella* and *Spirulina* sp. is a blue-green compound and widely used for its stable nature [142]. In contrast to chlorophylls, astaxanthin gives rise to strong red pigmentation, that are highly useful in most cosmetic products [155]. Phycoerythrin exerts intense pink fluorescence and is formulated for use in lipsticks, eye shadow, and other make-up essentials. Phycocyanin, in contrast, imparts blue fluorescence and is sourced from cyanobacteria (*Spirulina* sp. or *Arthrospira* sp.). In the Japanese cosmetic market, phycocyanin isolated from *Arthrospira* sp. has been commercialized as cosmetic colourants and eye shadow products [140].

Apart from being used as colourants, these microalgae-pigments are also utilised in tanning pills. Tanning is a process defined as the skin adaptation to UV exposure whereby the increased melanin level plays role in protecting the skin from sunlight rays that induce free radical formation [140,156]. Cantaxanthin, a pigment that is found predominantly in green microalgae (*Chlorella* sp., *Haemotococcus* sp. *Nannochloropsis* sp.) is one of the most common ingredients utilised in tanning pills. Canthaxanthin imparts its tanning effect by darkening the skin via deposition of its red-orange colour in the epidermis and subcutaneous fat. However, it is important to address that canthaxanthin tanning pills are yet to be approved by FDA where adverse effects of urticarial, hepatitis and fatal aplastic anemia have been noticed with daily consumption of these pills [140].

### 5.5. Whitening Agent

In contrast to tanning effect, the favourability towards fairer skin tone especially among Asian women has attracted much interest on whitening products as part of their beauty regime. Whitening effect of skin arises with the inhibition of tyrosinase enzyme, which plays vital role in melanin biosynthesis. Tyrosinase catalyses melanin synthesis via L-tyrosine hydroxylation to 3,4-dihydroxy-L-phenylalanine (L-DOPA) as well as oxidation of L-DOPA to dopaquinone followed by further conversion to melanin [157]. Several microalgal species are noted for their superior tyrosinase inhibition activity, such as *N. oculate* or *H. pluvalis*. Both zeaxanthin and astaxanthin pigments from these microalgae species are addressed for their anti-tyrosinase ability, making them suitable for cosmetic products intended for skin whitening [140,158,159].

### 5.6. Moisturising Agent

Moisturisation is a very critical step in preventing premature ageing of the skin, by maintaining its elasticity and radiance. The level of moisture retained within the human body is correlated with regulated water transport, corneocytes and hyaluronic acid as well as washing frequency [160,161]. In common practice, cosmetic range of products intended for moisturising effect is formulated with hydroxy acid however; this elevates the price range due to its limited supply. As such, microalgae-derived polysaccharides are preferred for its abundance as well as environmental-friendly nature. A review by Wang and colleagues highlighted the potential of an extract from *Chlorella vulgaris* that functions to support skin tissue along with collagen synthesis that assists in reducing wrinkle formation [161].

### 5.7. Anti-Inflammatory

Another vital and challenging issue of cosmetic products lies on the effect of neurogenic inflammation that imparts its implications of irritation and itching as the adverse effects. It is worthwhile to understand that most skin metabolic processes are affected by numerous signals in response to stress-induced brain nervous stimulus [162]. These signals include secretion of different hormones/substances from the various skin cells, such as indicated in Table 4 below.

In response to nervous stress, the neurotrophins signal becomes crucial for some inflammatory process (atopic dermatitis, psoriasis) where it induces proliferation of cutaneous nerve endings along with symptoms ranging from itchiness and pain [162,167,168]. Despite of the limited findings available on microalgae extracts implications on skin disorders, however, there are several bioactive compounds derived from microalgae that are shown to be effective. One such example is of the compound astaxanthin where it is vital in suppressing neuropathic pain in rats; where this involves inhibition of *N*-methyl-d-aspartate receptors that is also part of pain mechanism caused by neurogenic inflammation [162,169,170].

### 5.8. Industrial Applications of Microalgae in Cosmeceuticals

Microalgae-based products have gained much popularity, and are continuously ventured. Although the marketed products keep increasing over the years, yet it is still under par compared to its active ingredient potential. Most of the products are those focused mainly on antioxidant and photo-ageing protections; where there are still much more to be explored in terms of products intended for skin appendages treatment, modulation of fat adipokines as well as the skin microbiota [162]. At the same time, it is still important to highlight that it is economically disadvantageous in microalgae exploitation as an isolated active compound source due to the relevantly high cost of biomass production as well as purification [3,162,171,172]. Despite of these, continual recommendation and exploration to utilise microalgae-derived metabolites for the synthesis of products with multiple uses are inter-related with it being fully natural source along with its numerous benefits especially in cosmetic care [162,173].

In a review by Mourelle and colleagues, it is highlighted that several cosmetic-based industries have invested in establishing their own microalgae cultivation. These include renowned companies such as Louis Vuitton Moët Hennessy (Paris, France), Danial Jouvance (Carnac, France) and AGI Dermatics (America). One of the latest highlighted products in 2020 is the Argan Beta-Retinoid Pink Algae Serum launched by Josie Maran. This product is comprised of the main compound β-carotene extracted from pink algae that play vital roles to eliminate fine lines, wrinkles, dark spots, dullness, dry and flaky skin. The serum is also enriched with organic argan oil, quercetin and is claimed to be free of synthetic colours, fragrance, parabens, petrochemical or phthalates [174]. In addition, various other microalgae-rich extract products are also commercialized on a continual basis. Among these, several products available in the market are as shown in Table 5 below.

Apart from the commercialized products, a recent study in 2017 reported on the potential of a novel, non-fastidious freshwater microalgae (*Chlorella emersonii* KJ725233) for possible application in cosmeceuticals accounted by its anti-ageing, antioxidant and anti-inflammatory properties. Its ability to inhibit elastase, hyaluronidase, collagenase and to scavenge free radicals supports skin tissue that gives rise to skin rejuvenation. In addition, phytol compounds, shown to reduce the proteinases activity and thus, reduces the dermis inflammation [141].

## 6. Microalgae in Biofuels and Energy

Energy production has become the priority of the world’s population due to the fuel demand for transportation, electric power generation and operation of manufacturing plants. There are two groups of global energy sources; non-renewable (fossil) and renewable. The renewable energy sources which have been investigated to displace the depleting fossil energy sources include solar, wind, nuclear, geothermal, hydrogen, waves, tidal and algal biomass energy [182,183].

The first generation of biofuels comes from terrestrial crops (e.g., maize, sugarcane, sugar beet and rapeseed) which has many drawbacks/limitations causing destruction of forests and the abundant usage of water. The second generation is from forest residues, lignocellulosic agriculture and from non-food crop feedstocks which disadvantage is the land use [184,185]. Biofuels are the renewable energy sources produced from algal biomass which is the third generation of biofuels and is a promising alternative method to the previous methods. The advantages of using algae to generate fuels/energy are that it can be produced all-year-round, easy to cultivate in water without much effort, requires less water compared to terrestrial crops, does not require pesticides or herbicides thus reducing the cultivation cost [186,187].

**Figure 6:**
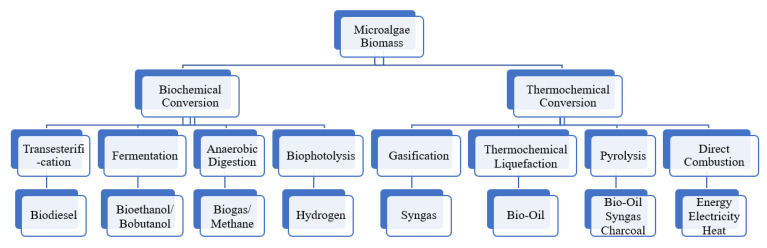
Various methods of producing biofuels/energy from microalgae biomass. Adapted from [16,188].

Alga biofuel has the potential to meet global demand since they do not compete with the production of food products. They can be exploited to biofuels inclusive of biodiesel, bioethanol/biobutanol, syngas, biochar, biogas and energy (electricity/heat) through various processing technologies such as thermochemical conversion (transesterification, thermochemical liquefaction, direct combustion, gasification and pyrolysis) and biochemical conversion (fermentation, anaerobic digestion and photobiological hydrogen production) (Figure 6) [16,188].

### 6.1. Biochemical Conversion

Biochemical conversion is the conversion of microalgae biomass to biofuels with the aid of biocatalysts, such as enzymes and microorganisms such as bacteria and yeast [189]. This method works well with high-water content biomass. The biochemical conversion includes transesterification, fermentation and anaerobic digestion.

#### 6.1.1. Transesterification-Biodiesel

Fatty acid methyl ester (FAME) called as biodiesel is produced by transesterification or esterification; a chemical reaction between alcohol and triacylglycerides without or with catalysts such as alkaline catalyst (sodium hydroxide or potassium hydroxide) or acid catalyst (sulphuric, sulphonic, phosphoric, and hydrochloric acids) [190,191]. Many species of microalgae such as *Scenedesmus obliquus, Neochloris oleabundans, Nannochloropsis* sp. and *C. vulgaris* contain a high content of lipids including triacylglycerides that are needed for the biodiesel production [192,193]. The production of biodiesel involves several stages including lipid extraction using solvents; methyl or ethyl alcohol (unrefined lipid), followed by lipid recovery with centrifugation-solvent evaporation (purified lipid and free fatty acids), transesterification or esterification (biodiesel production). The final step of this process is the removal of the solvent for the biodiesel recovery [193]. Biodiesel from renewable resources such as microalgae has several advantages compared to petroleum diesel; non-toxic, biodegradable, renewable and contains low levels of carbon monoxide, soot, hydrocarbons and particulates. It also discharges a low level of CO_2_ of up to 78% lower [194]. Many researchers have look into the possibility of producing biodiesel alongside with bioethanol from algae biomass. A study by Wang et al. [195] had shown encouraging results of *Tribonema* sp. with enriched lipid and carbohydrate content for biodiesel and bioethanol production. The conversion rate of lipid extracted with hexane-ethanol to biodiesel was 98.4% and the maximum yield of ethanol using yeast *Saccharomyces cerevisiae* was 56.1%.

#### 6.1.2. Fermentation-Bioethanol

The chemical and enzymatic pre-treatment method are usually used in the production of bioethanol. The common strong acids used include hydrochloric, sulfuric and nitric acid, whereas enzymes such as amylases, cellulases and invertases are used. However, using enzymes can be rather expensive if involves large scale production [196]. Sometimes the cell walls are disrupted using a mechanical method such as by 24/24 freezing/defrosting cycle to release the intracellular substances [197]. The fermentation of the treated biomass is usually using yeast *Saccharomyces cerevisiae* or bacteria at warm temperature (30–40 °C) to obtain ethanol and a bioreactor is later used for up-scaling [198,199]. The quality and quantity of the produced bioethanol are fully determined by the method used and fermentation process parameters inclusive of pH, temperature and the fermenter organism used [200].

Previous studies have shown that several microalgae species can act as an effective feedstock for the production of bioethanol. The species include *Chlorococcum humicola, Scenedesmus abundans PKU AC 12, Chlorella vulgaris* FSP-E, *Scenedesmus obliquus* CNW-N, *Porphyridium cruentum, Desmodesmus* sp., *Spirulina platensis* and *Scenedesmus acuminatus* (Refer Table 6). A comparison study of red microalgae (*P. cruentum*) culture conditions for bioethanol production was done by Kim and co-workers in 2017 [201]. The results indicated ethanol conversion yields from *P. cruentum* cultured in freshwater was much higher (70.3%) than the *P. cruentum* cultured in seawater (65.4%). Recently, Chandra et al. [202] study the effect of the cultural variables on the carbohydrate accumulation of *Scenedesmus acuminatus* to produce bioethanol. The bioethanol yield obtained was 0.12 g/g with the supplementation of lysine in the medium culture and at higher initial culture pH (pH 9.0).

#### 6.1.3. Anaerobic Digestion-Biogas/Methane

Microalgae can be converted to biogas through anaerobic digestion which is a biochemical process that mineralizes organic material through the action of microorganisms in the absence of oxygen [210]. Biogas is mainly made up of a mixture of methane, CH_4_ (50–70%), CO_2_ (30–45%) and traces of other gases such as hydrogen, H_2_ (<2%) and hydrogen sulphide, H_2_S (<3.5%). The process involves three stages; hydrolysis, acetogenesis/acidogenesis and methanogenesis [211]. Biogas production is often reduced due to the thick and rigid microalgae cell wall, therefore it is normally incorporated with production of other bioproducts such as biodiesel or bioethanol [212]. To overcome the cell wall problems, pre-treatment is usually done to enhance the process. Pre-treatment with heat was found to enhance the production of biomethane from *Chlorella* sp. by 11% as the temperature was increased to 90 °C from 70 °C for 0.5 h compared to the control [213].

#### 6.1.4. Biophotolysis-Hydrogen

Green microalgae and cyanobacteria can produce hydrogen through biophotolysis process. They possess chlorophyll a and the photosynthetic systems; Photosystem II (PS II) and Photosystem I (PS I), that enables them to perform photosynthesis by absorbing solar energy and converting water into hydrogen and oxygen [214,215]. Biophotolysis can be divided into two; direct and indirect pathways. During direct biophotolysis, water splitting at PS II generated electron and proton, giving rise to H_2_ in both green algae and cyanobacteria. For indirect biophotolysis, the degradation of carbon compounds will generate protons and electrons for the production of hydrogen which are mostly found in cyanobacteria [216].

There are several green microalgae and cyanobacteria such as *Tetraspora* sp. CU2551, *R. rubrum, R. spheroides, Rhodobacter capsulatus, C. vulgaris, C. reinhardtii, Anabaena* sp. and *Nostoc* sp. which have been widely studied for the hydrogen production [217,218,219,220]. The model microalga that is usually used for research is the *C. reinhardtii* since some of its organelles such as mitochondrial, nuclear genomes and chloroplast have been successfully sequenced [221].

### 6.2. Thermochemical Conversion

Thermochemical conversion is a process whereby microalgae biomass is converted to biofuels with the involvement of heat at a different temperature depending on the final product desired. This method can be used for both dry and wet biomass. Normally, in the thermochemical conversion no chemicals are added and the period for producing biofuels from this method is shorter than the biochemical conversion [222]. The thermochemical conversion methods include gasification, thermochemical liquefaction, pyrolysis and direct combustion.

#### 6.2.1. Gasification-Syngas

Syngas or synthesis gas is mainly composed of methane, hydrogen, carbon monoxide and carbon dioxide that can be used to generate heat or electricity [182,223]. Microalgae biomass is converted to Syngas through a gasification process which utilises partial oxidation with a mixture of oxygen and steam at high temperatures (>700 °C) [224].

#### 6.2.2. Thermochemical Liquefaction-Bio-Oil

Crude bio-oil is produced by thermochemical liquefaction in the presence of a catalyst at a temperature between 300–350 °C [225]. Hydrothermal liquefaction is the preferred method for wet algae biomass which uses water and elevated pressure [224]. The thermochemical liquefaction yield of microalgae depends on various parameters including type of catalysts and reaction temperature [226].

#### 6.2.3. Pyrolysis-Bio-Oil, Syngas, Charcoal

Bio-oil, syngas and charcoal can be produced through pyrolysis; the conversion of microalgae biomass in the absence of oxygen at medium to high temperatures (400–600 °C) [227]. Different products produced at different stages of temperature and duration of the process. Bio-oil is produced with a moderate temperature (500 °C) and a short-time exposure to vapour known as flash pyrolysis. Syngas is produced through fast pyrolysis at moderate temperature (500 °C) and moderate time exposure to vapour. Charcoal is produced through slow pyrolysis at a lower temperature (400 °C) and long-time exposure to vapour [228].

#### 6.2.4. Direct Combustion-Energy (Heat/Electricity)

Direct combustion is the burning of the microalgae biomass to generate heat, energy or electricity. It is normally done in a boiler or steam turbine at a temperature above 800 °C in the presence of oxygen. The process can produce 300 MW for domestic or a large-scale industrial process [16]. However, the overall operation cost may increase due to the pre-treatment requires by the microalgae biomass such as drying [229].

### 6.3. Industrial Applications of Microalgae in Biofuels

The potential of microalgae to be used as a source of biofuel in the future to replace the fossil fuel has always been a concerned [230]. However, there are several challenges that need to be addressed to enable success which include the development of low-cost, effective cultivation systems, efficient and energy-saving harvesting techniques because there are problems to discard large volumes of water during harvesting and methods for oil extraction and conversion that are environmentally friendly and cost-effective by selecting the best strain for higher production of biofuel [183,231]. In terms of cost, the harvesting process may involve a lot of money and commercialization will be too expensive as reported by the U.S. Department of Energy (DOE) who has been doing research in this area for about 16 years from the year 1980s to 1990s [232]. The only oil major company known to be still investing in the microalgae biofuel project is the ExxonMobil. In 2017, after 8 years of collaboration with a biotechnology company Synthetic Genomics, they announced a major breakthrough in producing microalgae with 40 percent more lipids while maintaining growth rates using CRISPRCas9 genome editing technique [233]. However, to date no recent developments are known regarding this issue. Although the microalgae-based biofuel production could not be commercialised in near future, there is still a possibility of using microalgae for biofuels in the future because of the abundance of resources such as the significant amount of land, water and CO_2_ available to support the algal biofuel technology [232]. Lately, algal biofuel production has attracted interest again from many researchers to explore in this field to be commercialised later. The areas of study and development that need to be considered to achieve success are in the cultivation, harvesting and extraction methods where the growth rate of microalgae need to be enhanced with a more robust alga growing systems, new species/strains with high lipid content need to be sought or the lipid content in the microalgae needs to be enhanced in various ways which are found to be appropriate and the development of an efficient method for the biofuel extraction needs to be explored so as not to increase the production cost [232].

## 7. Microalgae in Biofertilisers

Biofertilisers are natural substances or products containing live microorganisms that enhance the chemical and biological properties of the soils, revive soil fertility and stimulate plants growth [234]. Plants need nitrogen to grow and the lack of such component can be overcome by giving fertiliser at an adequate rate. However, excessive and prolonged use of chemical or synthetic fertilisers resulted in environmental pollution that will eventually cause an imbalance in the ecosystem [235]. As an alternative, microalgae have been extensively studied to see its potential as plant biofertilisers and also biostimulants.

The majority of cyanobacteria can fix nitrogen from the atmosphere and several species are known to be efficient as cyanobacterial-based biofertilisers such as *Anabaena* sp., *Nostoc* sp. and *Oscillatoria angustissima* [236,237,238]. Some of the various green microalgae and cyanobacteria species successfully used as biofertilisers to enhance crops growth include *Acutodesmus dimorphus, S. platensis, C. vulgaris*, *Scenedesmus dimorphus, Anabaena azolla and Nostoc* sp. (Table 7), with *Chlorella vulgaris* as one of the most commonly used microalgae in biofertiliser studies. The germination of *Hibiscus esculentus* was accelerated using combined seed and soil treated with *C. vulgaris.* Significant improvement of the soil nutrient content and microorganism count before treatment with the biofertiliser was also observed [239].

The treatment using green microalgae/cyanobacteria have shown many beneficial effects on the plants and soils. The seed germination, plant growth, yield and the nutritional value of the crops is enhanced besides the improvement of the soil fertility. The carbon and organic content of the soil were accelerated due to the excretion of carbon (exopolysaccharides) by the green microalgae/cyanobacteria into the soil and the degradation of the biomass and grazing activity add on to the increment [248,249,250,251]. Studies had shown that those factors influence the microbial activity and biomass of other microflora and fauna in the soil which eventually will stimulate the growth of the crops [248,252].

A study by Bumandalai et al. [244] exhibited the potential of *Chlorella vulgaris* as biofertiliser for the germination of tomato and cucumber seeds. The length of the tomato and cucumber roots and shoots were improved using algal suspensions of 0.17 and 0.25 g/L, respectively. Treatment of plants with *A. dimorphus* biofertiliser before seedling transplant showed enhanced germination, increased production of branches and flowers compared to the control group and the treatment group applied during transplant [240]. Nayak et al. [245] successfully used de-oiled microalgal biomass of *Scenedesmus* sp. as biofertiliser to improve the growth of rice plant.

The cyanobacteria can colonize different parts of a plant tissue such as in the roots and shoots, stimulating the nitrogen fixation and phosphorus solubilising microbial population on that parts, thus enhancing and improving the growth, nutritional status and defence mechanism of the plant and soil fertility [253,254,255,256,257,258]. Apart from that, cyanobacteria also produce siderophores (organic compounds) to facilitate in chelating micronutrients (e.g., Fe and Cu), known as biomineralization to make them easily accessible for plants growth [259,260].

Many green microalgae/cyanobacteria are also reported to excrete intracellular hormones to their surroundings which help to stimulate plants growth [261,262,263]. Examples are the green microalgae, Chlorophyta and Cyanophyta [264] and some cyanobacteria strains which produce cytokinins and auxins which help to stimulate plants growth parameters such as shoot length, root length, spike length and weight of seeds [265].

The use of green microalgae/cyanobacteria as biofertiliser can increase the activity of defence mechanism of the plants and improve their immunity by increasing the plant RNA activity, producing nutrient assimilating enzymes; dehydrogenase, nitrate reductase, acid or alkaline phosphatase, generating antioxidant and plants defence enzymes such as peroxidase, polyphenol oxidase, phenylalanine ammonia lyase and β-1, 3-endoglucanase in root and shoot of the plants [256,266,267]. Different strains of microalgae might provide different levels of defence mechanisms as shown by a study by Babu et al. [268]. Three different cyanobacteria (*Anabaena laxa* RPAN8, *Calothrix* sp. and *Anabaena* sp. CW2) inoculated in wheat plant showed the highest activity of peroxidase, polyphenol oxidase and phenylalanine ammonia-lyase with *Calothrix* sp. The results could suggest that using a combination of different types of microalgae with different abilities as biofertiliser, may contribute to the increase of plants immunity.

Some researchers had tried using a mixture of different microalgae species or a combination of microalgae and other organic or chemical fertilisers to further enhance its effectiveness. Maize plant treated with *C. vulgaris* and *S. platensis* along with cow dung manure for 75 days under greenhouse conditions, appeared to improve the growth and yield of the maize plant [241]. Jochum et al. [245] utilised a mixed culture of *C. vulgaris* (UTEX 2714) and *Scenedesmus dimorphus* (UTEX 1237) biomass as biofertiliser for rice plant. The biofertiliser showed efficacy with a significant increase in height of the rice plant. Growth parameters of onion plants were found to enhance with the application of a mixture of *S. platensis* and *C. vulgaris* showing higher growth rate and yield compared to the control group [243]. Consortia of green microalgae and cyanobacteria also showed promising results with the improved activity of soil microbial, increase in soil organic carbon, macro- and micronutrients and enhanced plant growth and yield [269,270].

### Industrial Applications of Microalgae Biofertiliser

The cost of using chemical fertilisers are increasing every year due to the high demand for foods from plants since it is one of the major important components for plant growth, especially for food production. Microalgae-derived biofertiliser can be an alternative for chemical fertiliser and has gained more popularity due to the natural effects they provide to the crops and the surrounding soil. The chemical fertiliser may cause several problems, among them are the pollution (soil, water, air) to the environment, reducing input efficiency, decreasing food quality, developing resistance in weeds, diseases and insects, micronutrient deficiency in the soil, degradation of soil and causing toxicity effect to different beneficial organisms living in the soil [271]. On the other hand, the biofertilisers are eco-friendly, more economical and more efficient [235]. Several things that are considered before the commercialisation of microalgae as biofertiliser are the formulation of the inoculate, the nature application of the products, packaging issues and also products storage [272]. Several countries have successfully commercialised the microalgae-derived biofertiliser products, which include the developing and developed countries. Previously in 2019, a Spanish company, Biorizon Biotech, S.L. had applied a patent for the method of obtaining concentrates of biofertilisers and biostimulants for agricultural use from green microalgae and cyanobacteria biomass [273]. Among the available microalgae-derived biofertiliser products in the market are as shown in Table 8.

## 8. Microalgae in Wastewater Treatment

Microalgae can utilise nitrogen, phosphorus, carbon and chemicals such as heavy metals from waste water as their source of nutrients and this is called as phycoremediation [280]. Conventional wastewater treatment plants (WWTPs) is designed basically to remove organic matter and nutrients. Nitrogen (N) and Phosphorus (P) are the most important pollutants in the aquatic environments because they are discharged into water bodies in huge quantities. Removal of N and P using microalgal remediation technology has high removal efficiencies which N and P contaminants can be completely removed from WWTPs, lower costs operation, zero sludge generation and high-value products can be generated such as fatty acids, pigment, biomass, biodiesel and unicellular protein [281,282,283,284].

New technology in removing heavy metals such as zinc, copper, lead, mercury, chromium and cadmium from the wastewater (WW) is by using microalgae biomass. Microalgae biomass is an option owing to low obtaining costs and close to 100% efficiency in the removal of heavy metals [285,286]. Microalgae also able to remove colorant such as aniline used by textile, cosmetics and food industries. A major portion of colorant removal is due to the interaction of the molecule with the reactive sites of the cell wall which permits the elimination of the colorant in a rapid manner [287,288].

Emerging contaminants (ECs) are molecules produced in industries such as pharmaceutical (used for human beings and livestock) and chemicals, including compounds such as hormones, antibiotics, plasticizers, antipyretics, antifungal and surfactants which cannot efficiently remove by WWTPs [289,290]. The removal of ECs is normally done by physicochemical processes such as photo-oxidation, ozonisation, or oxidation via metallic catalysis. Algae-based bioreactors have gained special research interest as a promising way to remove pharmaceuticals-based ECs from the wastewater either partially or completely. A wide spectrum of technologies has been used for the removal processes for ECs, mainly grouped as physical adsorption, chemical advanced oxidation and biological degradation processes and combination. Laboratory scale (open pond and bubble column photobioreactor) showed high removal percentages have been reached for ciprofloxacin, metoprolol, triclosan, and salicylic acid (>90%); moderate removal for tramadol and carbamazepine (50–90%), and trimethoprim and ciprofloxacin show fewer promising results with very low removal (<10%) [291].

Based on the latest research, phenol and its derivatives could be metabolized and degraded by algae. The ability of algae to remove phenolic compounds dependant with the type of algae or substrate, degradation time and initial pH. However, algae have a certain tolerance range to phenol [292,293]. Antibiotics are other pollutants that have been released to the environments and create antibiotics resistance. Recently, microalgae-based technology has been explored as a great potential alternative for the treatment of wastewater containing antibiotics by adsorption, accumulation, biodegradation, photodegradation, and hydrolysis [294]. Two freshwater algae species *Scenedesmus obliquus* and *C. pyrenoidosa* were shown to have the capability to transform steroids such as progesterone and norgestrel [295].

A device called Microalgae-microbial fuel cell (m-MFC) integrated process has been developed to overcome the problem of fossil-fuel depletion and environmental pollution by generating electrical energy from wastewater and sunlight, wastewater treatment, CO_2_ sequestration and biomass production in single, self-sustainable technology [296,297]. Table 9 showed microalgae used in the remediation of the pollutants present in the wastewater.

## 9. Microalgae in Feed

Microalgae biomass have also been successfully employed in feed formulations for different animals like cattle, fish, goat, lamb, poultry, pigs and rabbits. In aquaculture, species of genus such as Nannochloropsis, Isochrysis, Pavlova, Phaeodactylum, Chaetoceros, Skelotenma, Thalassiosira and Tetraselmis are used as biomass [310]. A microalgae, *D. salina* has been applied in animal feed for its rich protein (57% d.w.) and carbohydrates (32% d.w.) contents [311]. Microalgal based feed affects the animal’s physiology as well as it improves its immune response and fertility. The combination of 5–15% of algal biomass, mixed with animal feed, can be used safely as a partial replacement for conventional proteins. However, the use of higher concentrations of microalgae biomass results in less feed intake by some animals due to the lower palatability of the feed. The quality of proteins with microalgal sources have a comparable or superior functionality (either as foam, emulsifier, solubilizer, surfactant, or gelling agent) equal or even greater than other commercial protein sources [312]. Chlorella biomass showed that it can be digested easily up to 5% in the form of paste [44]. Microalgae can provide an important source of essential fatty acid to fish. DHA and EPA are not synthesized by fish and the only resource to enrich them in fish are by accumulating them in proper algal paste and store them as fish feed [313,314]. Microalgae, like *C. vulgaris* and *A. platensis*, consist of amino acid profiles that show similarities to soybean which is currently the main source of protein used in feed. In addition, the microalgal biomass is a potential source of minerals; *C. vulgaris*, *A. platensis*, *Micractinium reisseri*, *Nannochloris bacillaris*, and *Tetracystis* sp. showed very high contents of iron in contrast to soybean [315].

## 10. Microalgae in Proteomics

Genetic engineering has been used to manipulate microalgae which allow researchers deducing metabolic pathways in order to construct new algal metabolism methods. This will enable to produce new molecules useful for biotechnological applications [316]. Microalgae proteomics can give the information of how genes of interest are being expressed as proteins which will help researchers to find proteins with biotechnological applications such as anti-inflammatory proteins [317].

The potential of microalgae in the production of proteins with biotechnological application has been explored. *C. reinhardtii* has been extensively studied for therapeutic protein production and more than 100 such proteins have been expressed successfully in this system [318]. Microalgae-based biomanufacturing is preferred due to effectiveness in terms of cost and energy, fewer contamination chances, and simplified downstream processing. Malaria detection protein which are used in ELISA testing and cell-traversal protein as an antigen from sporozoites and ookinetes of mosquitoes have been successfully expressed and produced in *C. reinhardtii* chloroplast [319]. Phycobiliproteins from cyanobacteria and red algae have been described to present antioxidant, hepato-protective, anti-inflammatory, immune-modulating, and anticancer effects [48,320,321,322]. *Chlorella pyrenoidosa* and *Chlorella vulgaris* have shown anti-hypertensive and anti-tumour activities from its isolated peptides [322].

A protein isolated from *Chlorella sorokiniana* has high-value bioactive peptides with nutraceutical and pharmaceutical application, using proteomics techniques [323]. Most of the proteomics approaches in the area of microalgae research are focused in development of efficient biofuels production [324,325,326,327].

## 11. Conclusions

Microalgae are promising source of generous amount of metabolites with essential health benefits. Continual exploration for novel microalgae species along with isolation of its bioactive compounds are in great demand for diverse applications in various fields and industries as raw material, biomass or high-quality extracts. Despite of the excellent and superior benefits, there are several limitations that should be addressed as well. The significantly high incurring cost of the isolation might result in over-priced products, where it is believed this could be partly subsidised with utilisation of single solvent to generate high extract yield. In addition, it is essentially important to highlight that these are natural extracts with unique composition and varies subject to the season, cultivation condition, as well as the extraction method. Studies are also focusing on extraction of more than one product from a single biomass for a cost-efficient industry. On the other hand, the process of bringing a drug candidate to the market requires extensive pre-clinical testing and clinical trials to determine the safety and efficacy of the drug before it is approved by the FDA and is a very costly and time-consuming process. Hence, not all potential bioactive will eventually reach the market and consumers. Indeed, low yield of bioactive and strenuous and expensive purification steps have been regarded as the main challenge in producing economically viable drugs from microalgae. Therefore, to overcome these issues some of these active compounds are currently expressed in suitable vectors to ease purification and hence reduce the purification and production costs. Efforts on cost reduction by studying effect of different culturing conditions such as light intensity, nutrient availability and harvesting time on the metabolite yield and bioactivity as well as producing high-yield microalgae species via genetic modification approaches are currently pursued to accelerate the commercialization process of some of these bioactives. Meanwhile, advancements in biotechnology applications can help overcome some issues of poor oral bioavailability or instability in the gastrointestinal tract of some microalgae bioactives like peptides and fatty acids. This includes novel approaches using encapsulation technique and nano formulation to improve the solubility and bioavailability of some microalgae derived compounds for treatment of diseases. Furthermore, genetic engineering approaches expressing microalgae metabolites in natural organism for drug delivery have been explored. Nevertheless, more studies especially in in-vivo models and clinical trials are still needed to determine the safety and efficacy of these novel drugs prior to drug approval and commercialization.

In conclusion, it is vital that more efforts should be taken in developing multi-functional range of products, that are affordable and is inter-related with nutritional science that could cater for even more health benefits comparatively to the ones in current market. These findings would bring much more insights into the vast potentials of microalgae-derived metabolites that remain to be explored and assessed. Further roadmap towards enhancing phycoeconomy is recommended by uplifting the current biological and technology process by taking into account on sustainability and environmental benefits.

## Figures and Tables

**Table 1:** Important micro-algae derived components in pharmaceutical applications.

Compound	Microalgae Species	Isolation/Extraction Method	Cell/Virus/Animal Model/Clinical Patient	Effect	References
Anticancer Agents					
Lutein	*Chlorella vulgaris*	Ethanol extraction and partitioned with hexane	Colon cancer (HCT116) cells	Antiproliferative effect IC_50_ = 40.31 ± 4.43 µg/mL	[31]
Violaxanthin	*Dunaliella tertiolecta*	Dichloromethane extract	Human mammary carcinoma cell lines (MCF-7) and human prostatic carcinoma (LNCaP) cells	Potent inhibition of MCF-7 and LNCaP cells at growth inhibition (GI_50_) of 56.1 and 60.9 µg/mL, respectively	[32]
Fucoxanthin	*Phaeodactylum tricornutum* UTEX 640	ressurized liquid extraction	Human liver cancer cell lines (Hep-G2), Human colon cancer (Caco-2) cells and HeLa cell line	An inhibitory effect of up to 58% was measured in Hep-G2 cells. In HeLa and Caco-2 cells, the effect was stronger than that of the positive control with a final concentration of 5% DMSO.	[33]
Phycocyanin	*Spirulina platensis*	Freeze-thawing followed by solvent extraction	Liver cancer (Hep-G2) cancer cells, Non-small cell lung cancer (NSCLC)	Inhibited liver cancer cells and leukemia cells. Induce apoptosis in H460 cells reaching 3.72 ± 0.98% and suppress growth of NSCLC cells	[34,35,36,37]
Phycocyanin	*Spirulina platensis*	Supercritical fluid extraction	Human lung cancer cells (A549)	IC_50_ = 26.82 µg/mL	[38]
Phycocyanin	*Limnothrix* sp. 37-2-1	Fractional precipitation and purification with activated charcoal and chitosan	Prostate Cell Line LNCaP	C-PC alone (250 and 500 µg/mL) killed 65% and 70% of cells while C-PC (500 µg/mL) combined with Topetencan (TPT) (1 µM) killed 80% of cells due to additive effect	[39]
Phycocyanin	*Limnothrix* sp. NS01	Four-step purification procedure including the adsorption of impurities with chitosan, activated charcoal, ammonium sulfate precipitation, and ion-exchange chromatography	Human breast cancer cell line (MCF-7)	IC_50_ for 24, 48 and 72 h exposure to C-PC were 5.92, 5.66 and 4.52 µg/µL	[40]
Cardioprotective agents					
cis β-carotene	*Dunaliella bardawil* (containing a mixture of cis and trans-isomers)	Enriched extract	Mice fed a high-fat diet	In old mice with established atherosclerotic lesion, Dunaliella inhibited significantly plasma cholesterol elevation and atherosclerosis progression	[41]
Eicosapentanoic acid (EPA)	Nannochloropsis	Enriched EPA oil	Healthy subjects	Lower cholesterol levels	[42]
Antiviral agent					
Cyanovirin	*Nostoc ellpsosporum*	Aqueous extraction	HIV-1 laboratory strains; HIV-1 primary isolates; HIV-2; SIV;	HIV-1 laboratory strains (EC_50_ 0.1–5.8 nM), HIV-1 primary isolates (EC_50_ 1.5–36.8 nM), HIV-2 (EC_50_ 2.3–7.6 nM), SIV (EC_50_ 11 nM)	[43]
Scytovirin	*Scytonema varium*	Aqueous extraction	HIV-1 laboratory strain (HIV-1_RF_); HIV-1 primary isolates (ROJO) in peripheral blood mononuclear cell (PBMC); HIV-1 primary isolates (Ba-L and ADA) in macrophages	HIV-1_RF_ (EC_50_ 0.3 nM), HIV-1 primary isolates (ROJO) in PBMC (EC_50_ 7 nM), HIV-1 primary isolates (Ba-L) (EC_50_ 22 nM), HIV-1 primary isolates (ADA) (EC_50_ 17 nM)	[44]
Mixture of Icthypeptins A and Icthypeptins	*Microcystic ichthyoblabe*	Methanol extract	Influenza A	IC_50_ 12.5 µg/mL	[45]
Calcium spirulan	*Spirulina platensis*	Aqueous extraction	HIV-1; Herpes simplex virus type 1 (HSV-1)	HIV-1 (IC_50_ 9.3 µg/mL), Herpes simplex virus type 1 (HSV-1) (IC_50_ 9.0 µg/mL).	[46]
Sulfated exopolysaccharide	*Porphyridium cruentum*	Aqueous extraction	Herpes simplex virus type 1 (HSV-1); Herpes simplex virus type 2 (HSV-2); Vericella virus (VZV)	HSV-1 (CPE_50_ protection 1 µg/mL), HSV-2 (CPE_50_ protection 5 µg/mL), VZV (CPE_50_ protection 0.7 µg/mL).	[47]

**Table 2:** Examples of bioactive components from microalgae that are produced on a commercial scale.

Microalgae	Bioactive Component	Product	Pharmaceutical Applications	References
*Haematococcus pluvialis*	Astaxanthin	Spirulina (Earth Spirulina Group, ES Co, Seoul, Korea)	Lipid lowering	[53]
*Schizochytrium limacinum*	Docosahexaenoic acid (DHA)	Maris DHA oil (IOI, Hamburg, Germany)	Rheumatoid arthritis	[54]
*Nannochloropsis*	Eicosapentaenoic acid (EPA)	Almega^®^PL (iWi Life) (Qualitas Health, Houston, US)	Cholesterol lowering	[42]
*Arthrospira FEM-101*	Allophycocyanin	ApoX surface antiviral spray (FEBICO, Taiwan)	Antiviral	[55]

**Table 3:** List of commercialized strain, industry application, companies, and the countries involved.

Biomass	Extracted Component	Isolation/Extraction Method	Application in Industry	Company	References
*Spirulina platensis*	Algae protein; Biomass	Enzymatic hydrolysis, Physical processes, Chemical extraction, Ultrasound-assisted extraction, Pulsed electric field, and Microwave-assisted extraction	Health supplement, Health food, Infant formula	Saxony-Anhalt (Germany); Parry Nutraceuticals (India); Japan Spirulina Co., Ltd. (Japan); Siam Alga Co., Ltd. (Thailand)	[3,64,65]
*Spirulina platensis*	Vitamin 12	Water extraction method	Health supplement	Myanmar Spirulina Factory (Myanmar)	[65,66]
*Chlorella vulgaris* *Chorella* sp.	Biomass, pigments	Microwave-assisted extraction	Health food, food supplement	Nikken Sohonsha Corp. (Japan); Chlorella manufacturing and Co. (Taiwan); Klötze (Germany); Ocean Nutrition (Canada)	[3,65]
*Haematococcus pluvialis*	Astaxanthin	Mechanical treatments, chemical treatments using solvents, pressurized extraction, ultrasounds and microwaves	Food supplement, bio-colorant	Algae Health Science (China); Cyanotech Corporation (USA); Aquasearch Algatechnologies (Israel)	[65,67,68,69,70]
*Dunaliella salina*	Beta-carotene	Solvent extraction; Microwave- assisted extraction	Health food, Dietary supplement, bio-colourant	Cyanotech (USA); Earthrise Nutritionals (USA); Nature Beta Technologies Cognis (Israel); Betadene (Australia); Nature Beta Technologies Cognis (Australia)	[65,71]
*Spirulina platensis*	Phycocyanin	Microwave-assisted extraction	Bio-colourant, Health supplement	Panmol/Madaus (Austria); Yunnan Green A Biological Project Co., Ltd. (China).	[65]
*Ulkenia* sp.	DHA	Solvent extraction and microwave	Dietary supplement;	Nutrinova (Germany)	[3]
*Schizochytrium* sp.	DHA	Cellular hydrolysis	Dietary supplement; Health food	OmegaTech (USA)	[3]
*Crypthecodinium cohnii*	DHA	Microwave	Infant formula	Martek (USA)	[72]
*Nannochloropsis oculata*	Omega-3 PUFA	Solvent extraction; Microwave	Omega-3 supplements	Qualitas (USA); Cleanalgae SL (Spain); Astaxa (Germany)	[73,74]
*Porphyridium* spp.	Polysaccharides	Ultrafiltration	Food additives, Nutrition	InnovalG (France)	[65,75,76]
*Euglena gracilis*	Biomass	Flocculation	Health food	Euglena (Japan)	[77]
*Odontella aurita*	Fatty acids		Health supplement	InnovalG (France)	[65]

**Table 4:** Anti-inflammatory secreted signals of the different skin layers.

Skin Cells	Secreted Compounds	Reference
Keratinocytes, melanocytes	Corticotropin-releasing hormone (CRH), adrenocorticotropic hormone (ACTH), catecholamines	[162,163,164]
Dermal fibroblasts	ACTH, cortisol, prolactin	
Skin nerve endings	Adrenaline, noradrenaline, substance P	
Sebaceous glands	CRH, prolactin	
Cutaneous nerve endings and almost all skin cells	Produces and responds to special cytokines (neurotrophins)	[162,165,166]

**Table 5:** Selected representation of the commercialized microalgae-derived cosmetic care products.

Microalgae	Commercial Name/Company	Application	Reference
*Dunaliella salina*	Blue Retinol^™^	Stimulates skin cell growth and proliferation	[175]
*Nannochloropsis oculata*	Pentapharm	Promotes excellent skin-tightening	[142,175]
*Chlorella vulgaris*	Dermochlorella/CODIF Recherche and Nature	Stimulates collagen synthesis, supports tissue regeneration, reduces wrinkle formation, anti-ageing	[3,176]
*Arthospira* sp.	Protulines, Exsymol	Assists in skin-tightening and repairs signs of ageing skin	[144]
*Phaeodactylum tricornutum*	Givaudan	Prevents premature ageing	[142]
Argan Beta-Retinoid Pink Algae Serum	Josie Maran	Eliminate fine lines, wrinkles, dark spots, dullness, dry and flaky skin	[174]
*Porphyridium* sp.	Alguard^®^/Frutarom	Anti-wrinkle, protection from UV damage	[151,177]
*Porphyridium cruentum*	SILIDINE^®^/Greentech	Improves skin aspect, decrease redness, vascular toning	[142]
	Cicatrol^®^/Greensea	Anti-ageing, antioxidant	[178]
*Tetraselmis suecica*	O^+^ Gold Microalgae Extract^®^/Greensea	Anti-inflammatory	[178]
*Scenedesmus rubescens*	Pepha^®^-Age/DSM Nutritional Products	Protects from photo-ageing, pro-collagen	[179]
*Nannochloropsis oculata*	Pepha^®^-Tight/DSM Nutritional Products (Pentapharm)	Protects from oxidative stress, simulates collagen synthesis and skin tightening	[180]
*Dunaliella salina*	Pepha^®^-Ctive/DSM Nutritional Products (Pentapharm)	Stimulates collagen synthesis, cell proliferation, energy metabolism	
*Dunaliella salina, Haematococcus pluvialis*	REVEAL Color Correcting Eye Serum Brightener/Algenist	Treats uneven and dull skin, dark circles under eye, gives brighter complexion	[181]
*Chlorella vulgaris*	Phytomer/Phytomer	Protects skin, neutralises inflammation	[151]
*Chlorella* sp.	Golden ChlorellaTM/Terravia Holdings	Hydrates skin and hair	[142]

**Table 6:** Bioethanol production from various microalgae species.

Microalgae	Ethanol Yield (g Ethanol/g Substrate)	Reference
*Chlorococcum infusionum*	0.26	[203]
*Chlamydomonas reinhardtii*	0.24	[204]
*Chlorococcum humicola*	0.52	[205]
*Scenedesmus abundans PKU AC 12*	0.103	[206]
*Chlorella vulgaris* FSP-E	11.66 (87.59%)	[207]
*Scenedesmus obliquus* CNW-N	8.55 (99.8%)	[208]
*Desmodesmus* sp. FG strain SP2-3 (unidentified green microalga)	0.24	[209]
*Scenedesmus acuminatus*	0.12	[202]

**Table 7:** Microalgae species and their use as plant biofertiliser and biostimulant.

Microalgae	Crops	Reference
*Acutodesmus dimorphus*	Tomato	[240]
*Spirulina platensis*, *Chlorella vulgaris*	Maize	[241]
*Chlorella vulgaris*	*Hibiscus esculentus*	[239]
*Chlorella vulgaris* (UTEX 2714), *Scenedesmus dimorphus* (UTEX 1237)	Rice	[242]
*Spirulina platensis*, *Chlorella vulgaris*	Onion	[243]
*Chlorella vulgaris*	Tomato and Cucumber seeds	[244]
*Scenedesmus* sp.	Rice	[245]
*Anabaena azolla*	Rice	[246]
*Nostoc muscorum* *Nostoc rivulare*	Maize	[247]

**Table 8:** Commercialised microalgae-derived biofertiliser.

Microalgae	Commercial Name	Company	Country	Reference
Cyanobacteria (Blue-green algae)	Skipper Khad^®^ and Power Play-90 WSG	Ecological Products Industries	India	[274]
*Spirulina* sp.	Shwe Awzar^®^	June Industry Limited	Myanmar	[275]
*Spirulina* sp.	Algafert^®^	Biorizon Biotech	Spain	[276]
*Chlorella vulgaris*	Terradoc^®^	Mikroalg Food and Agriculture Industries	Turkey	[277]
Cyanobacteria	TerraSync^™^	Accelergy Corporation	USA	[278]
*Chlorella* sp.	EMEK	MCT Tarim Ltd.	Turkey	[279]

**Table 9:** Microalgae used in the remediation of the pollutants present in the wastewater.

Pollutants	Microalgae Bioremediation	References
Nitrogen & Phosporus	*Phormidium* sp., *Spirulina maxima*, *Chlorella vulgaris*, *Scenedesmus dimorphus*, *Scenedesmus quadricauda*, *Chlorella sorokiniana*, *Chlorella vulgaris ESP-6*	[281,282,283,284]
Heavy Metals	*Chlorella* sp., *Tetradesmus* sp., *Scenedesmus* sp., *Porphyridium* sp., *Chaetoceros* sp., *Oscillatoria* sp., *Sprogyra* sp., *Scenedesmus* sp., *Anacytis* sp., *Chlamydomonas* sp., *Anabaena* sp., *Ceratium* sp., *Scenedesmus* sp., *Calothrix* sp., *Arthrospira platensis*	[285,298,299]
Colorants	*Chlorella vulgaris, Cosmarium* sp., *Nostoc linckia, Spirogyra* sp.	[300,301,302,303]
**Emerging Pollutants**		
Antibiotics Phenol	*Nannochloris* sp., Mixture of algae-bacteria consortia in pilot high rate algae pond (HRAP), Mixture of algae-bacteria consortia (dominated by *Coelastrum* sp.) in HRAP, Mixture of algae-bacteria consortia in 1-L HRAP *S. obliquus*, *C. vulgaris, Chlorella* sp., *Scenedesmus* sp., *Chlamydomonas mexicana, Chlorella sorokiniana* *Chlorella vulgaris* *Chlorella pyrenoidosa*	[304,305,306,307,308] [293,309]

## Abbreviations

ACTH	Adrenocorticotropic hormone
ALA	α-linoleic acid
cGMP	current Good Manufacturing Practice
CPC	Chlorophytic polyculture
CRH	Corticotropin-releasing hormone
CV-N	Cyanovirin
DHA	Docosahexaenoic acid
DOE	U.S. Department of Energy
EAAI	Essential amino acid index
ECs	Emerging contaminants
EPA	Eicosapentaenoic acid
EAAI	Essential amino acid index
FDA	Food and Drug Administration
FP-PBR	Flat-plate open pond and closed photobioreactor
GRAS	Generally Regarded as Safe
HRAP	High rate algae pond
IC	Inhibitory concentration
L-DOPA	3,4-dihydroxy-L-phenylalanine
m-MFC	Microalgae-microbial fuel cell
PBR	Open pond and closed photobioreactor
PUFAs	Polyunsaturated fatty acids
ROS	Reactive oxygen species
SVN	Scytovirin
TPBR	Tubular open pond and closed photobioreactor
TAG	Triacylglycerides
TG	Triglyceride
UV	Ultraviolet
VCPBR	Vertical column open pond and closed photobioreactor
WW	Waste water
WWTPs	Waste water treatment plants
